# Comparative Analysis of the Stallion Field Performance Test at Different Training Stages and Horse Age

**DOI:** 10.3390/ani15223289

**Published:** 2025-11-13

**Authors:** Dorota Lewczuk, Alicja Borowska, Julia Andruszkiewicz, Emilia Bagnicka

**Affiliations:** 1 Institute of Genetic and Animal Biotechnology PASN Jastrzębiec, Postepu 36A, 05-552 Magdalenka, Poland; d.lewczuk@igbzpan.pl; 2 Faculty of Veterinary Medicine and Animal Science, University of Live Sciences, Poznań, Wojska Polskiego 28, 60-637 Poznań, Poland; alicja.borowska@up.poznan.pl (A.B.); julaa114@gmail.com (J.A.)

**Keywords:** performance test, stallion, age, training preparation

## Abstract

Breeding value estimations of Warmblood horses in Poland are based on field performance, but the amount of stallions’ data is still insufficient (334 horses). The first study’s aim is to compare stallions’ results in different stages and evaluate the overall ability using both stages. The effects on preselection (first stage free movement) and performance tests (second stage under rider) were analyzed separately and together as the overall ability. Spearman correlations were evaluated. The second aim was to evaluate the training period on the results. Due to the pandemic and lack of tests, horses had different training periods during the six years studied. The combined year-place effect, horse specialization group, and birth country had effects on four of twelve traits. The regression on age influenced only the preselection jumping trait (*p* = 0.04), while the training period influenced canter (*p* = 0.04) and “success” (passed/or not) in the performance test (*p* = 0.04). It seems that the training period and horse age are more significant for young horses’ performance. The correlations between stages for the same traits are moderate for gaits (<0.53) and low for jumping (<0.3). Thus, at least on a basic phenotypical level, results do not correspond strongly with each other.

## Simple Summary

The amount of data obtained since the beginning of the stallion field test in Poland is still insufficient to evaluate genetic value. However, the overall ability (traits of the same name treated together) could be analyzed to obtain more data, as the identify of significant factors influencing results is crucial. Therefore, the study aimed to compare stallion preselection and performance test results, as well as to assess impact of some factors: year, place, performance group, country of origin, age and training period. The research showed that associations between traits scored during two stages of selection were at a medium level. Analyses indicated that the performance group and country are the most important factors influencing performance results. The year and place were more significant for the first selection stage than for the second one. It was also found that that the training period is more significant for the performance results, like the age of horses in young horse groups. Therefore, it can be concluded that the horse data coming from different stages of stallion field performance tests, training, and horse presentation does not seem to be comparable. The preliminary analysis does not allow for the data to be combined for breeding purposes.

## 1. Introduction

Breeding value estimation is one of the most significant processes in animal breeding programs. Therefore, the detailed, objective assessment of performance value is crucial for the decision-making strategy. The current genetic improvement programs of Warmblood horses are carried out mainly in the broadly understood equestrian sport direction. The selection should be based on the most desirable traits that predispose a given individual to high sport achievements [1]. Therefore, the basic criteria for qualifying horses for breeding are the results of a performance test. Performance trials for young stallions and broodmares are held worldwide in the form of stationary or field performance tests [2,3]. In many countries, the breeding value evaluation of horses is officially carried out by national horse breeding associations, which use selection indices, calculated based on phenotypic assessments of horses [2,4,5,6,7,8]. In Poland, the most popular field performance tests for stallions involve preliminary qualifications for horses [9,10]. The stallions then undergo a 100-day training period leading up to the final test. Both events assess the horse’s free movement and jumping traits, while the final performance test also assesses the movement and jumping characteristics under the rider. Therefore, the comparison of the qualification results with the final performance test can give interesting information in the context of adding new information into the data file and doubling the information on horses for breeding value estimation. Comparable factors that may influence the results, as well as the relationships between them, can give preliminary information on using these preselection results in the breeding value estimation. High genetic correlations were obtained by Vilkund et al. [11] between corresponding traits in the 3-year-old test and the 4-year-old Riding Horse Quality Test; however, there are limited papers on the comparison of qualifications and final tests because this is due to the specificity of breeding programs in a given country, while some authors show genetic correlations between the traits assessed during breeding performance tests and subsequent results in sports [5]. Ducro et al. [12] showed that traits of movement assessment (walk, trot, balance, self-suspension) assessed during First Stallion Inspection showed negative genetic correlations with assessments of jump characteristics and results in jumping (up to −0.45). On the other hand, jumping traits had both genetic and phenotypic negative correlations with the results of dressage competitions. The Swedish linear evaluation was also used to predict sport success [13,14]. French researchers stressed the need for shortening the generation interval [15]; however, later studies go further to the introduction of genomic information [16,17,18]. Even if more sophisticated methods like innovative reproduction techniques (e.g., embryo transfer, sexed semen and embryos, cloning of geldings) can be used to maintain genetic progress [19], still, performance-based breeding value estimation is the most common breeding strategy [20,21,22,23,24,25,26,27,28].

The basis for assessing the breeding value of Warmblood horses in Poland is stationary and field performance tests. Unfortunately, the amount of data obtained since the beginning of the stallion field test introduced in 2017 is still limited (below 500) to estimate the genetic value or correlations. However, there is a need to characterize the recorded traits, identify the significant factors influencing them, and search for opportunities to obtain more data to assess the breeding value of horses. The study aims to answer the question of whether the stallion test data from different stages of training can be combined to allow quicker data collection for breeding value estimation and to evaluate the effect of the training period between preselection and performance tests on the final results.

## 2. Materials and Methods

The data on the 334 Warmblood stallions registered in the Polish Horse Breeders Association that took part in the preselection process for performance field test stations were collected. A total of 180 stallions qualified for the final stage of evaluation, and 144 were presented for the final test (8 were withdrawn due to health reasons). A detailed description of the number of horses in years is presented in Supplementary Table S1. Horses were comparable in conformation with a mean of 79 points (SD = 1.28; minimum = 78, maximum = 83). The size of stallions was uniform, with a mean height at withers of 168 cm (SD = 3.09; minimum = 161, maximum = 175), mean circumference of the chest of 188 cm (SD = 5.16; minimum = 165, maximum = 198 cm), and a circumference of the cannon of 21.2 cm (SD = 0.8; minimum = 19, maximum = 23). The age of horses was 1164 days on average (SD = 320) on the preselection stage and 1436 days (SD = 339) on the performance field test. Horses were evaluated in groups of performance origin (9% dressage, 75% jumping, and 16% eventing). Based on their specialty, horses were evaluated in gait quality (walk, trot, canter) and jumping. Additionally, type and conformation were evaluated. Only performance data were taken into analysis, and the success of the horse was treated as a 0–1 value. The performance data from the preselection consists of free walk, trot, canter, free jumping, evaluated by judges on a scale of 1–10 points (Table 1). The judge committee consists of three experienced judges, selected specially for stallion performance tests for longer periods. After discussions, the consulted mean of the three judges is used. The performance data are judged by the same commissions in the same scaling system for the following traits under the rider: walk, trot, canter, jumping, rideability, and trainability. Unfortunately, the dressage horses are not evaluated in jumping, and jumping horses are not evaluated in walk and trot. Thus, the number of observations is not equal for every trait (Table 1). Dressage and jumping traits on performance tests are evaluated under the rider who trained the horse throughout the whole test. The overall rideability and trainability in the specific discipline are evaluated by the outside rider, being the specialist in the equestrian discipline represented by the horse’s origin. The traits evaluated during both performance events are also presented in Table 1. Horse data were collected during six subsequent years of selection, and the COVID pandemic period was included in it. In this one-year COVID pandemic period, only qualifications could be organized, but not the final field test. That is why some horses had a longer period of training for preparation for the test. That allowed us to investigate the effect of the preparation/training period on the final performance test results. The training period was characterized as a mean of 346 days, with a standard deviation of 139 (minimum = 138, maximum = 700). The data were analyzed in different groups—preselection stage, field performance tests, and as the overall ability (the traits named comparable and treated as the ability without distinguishing free or under rider). There were three places for the qualifications (1—1%horses, 2—77%, 3—22%), and only the second one was available across 5 years of investigations. There were also three places for performance tests (1—23% horses, 2—38%, 3—39%) during the investigated years. The first one was available for the stallions only in the first year, but the others were available during 2 (second place) or 3 years (third place). Most of the horses originated from Poland (233), but also from Germany (37), the Netherlands (14), and single horses from Belgium, Ireland, Sweden, and Great Britain.

All performance traits were treated as normally distributed. The normality of the distribution was verified using the Univariate procedure (SAS/STAT, ver. 9.4; 2002–2012). Only the success trait, being binomial, was evaluated adequately, as it was not normally distributed. In order to compare results obtained on different stages of training, the following analyses were performed: analysis of variance to compare effects influencing results on different stages, and correlations between results on different stages. The overall skill ability, obtained as the mean for two stages of evaluation (free and under rider), was analyzed with the analysis of variance and a repeated model based on the sire’s effect. The analysis of variance for performance traits was performed using the GLM procedure of the SAS program (SAS/STAT, ver. 9.4; 2002–2012). For the binomial trait, the GLIMMIXED (SAS/STAT, ver. 9.4; 2002–2012) procedure was used. Spearman’s correlations were used for all traits to calculate their relationships. The Mixed and Glimmixed procedures were used for the repeatability calculations. The following effects were taken into account on the first preselection stage:

y_ijkl_ = YP_i_ + PG_j_ + CO_k_ + (β × age)_ijkl_ + e_ijkl_ (1)

where:

y_ijkl_—trait evaluation;

µ—mean of the trait;

YP_i_—fixed effect of year × place (i = 1, …, 7);

PG_j_—fixed effect of performance group (j = 1, 2, 3);

CO_k_—fixed effect of country of origin (k = 1, …, 6);

(β × age)_ijkl_—fixed linear regression on the age in days;

e_ijkl_—random residual effect.

The second-stage data, performance field test results, were analyzed using additional regression on the days of training/preparation:

y_ijkl_ = YP_i_ + PG_j_ + CO_k_ + (β × age)_ijkl_ + (β × training time)_ijkl_ + e_ijkl_ (2)

where:

y_ijkl_—trait evaluation;

µ—mean of the trait;

YP_i_—fixed effect of year × place (i = 1, …, 6);

PG_j_—fixed effect of performance group (j = 1, 2, 3);

CO_k_—fixed effect of country of origin (k = 1, …,6);

(β × age)_ijkl_—regression on the age in days;

(β × training time)_ijkl_—regression on training time;

e_ijkl_—random residual effect.

Finally, the overall ability of horses (evaluation for the same-named skills) was analyzed by the model:

y_ijklm_ = YP_i_ + PG_j_ + CO_k_ + E_l_ + (β × age)_ijklm_ + (β × training time)_ijklm_ + e_ijklm_ (3)

where:

y_ijklm_—trait evaluation;

µ—mean of the trait;

YP_i_—fixed effect of year × place (i = 1, …, 6);

PG_j_—fixed effect of performance group (j = 1, 2, 3);

CO_k_—fixed effect of country of origin (k = 1, …, 6);

E_l_—fixed effect of event (stage) (k = 1, 2);

(β × age)_ijklm_—regression on the age in days;

(β × training time)_ijklm_—regression on training time;

e_ijklm_—random residual effect.

The repeatability for the horse abilities was calculated using the same model (3) with the random effect of the sire (209 sires) in Mixed and Glimmixed procedures in SAS. The LSMs were calculated using a post hoc Tukey test for event effect in the analysis of variance for ability traits.

## 3. Results

The results of two stages of training showed that the performance group and country of origin are the most important factors influencing performance results (Table 2). However, these effects were of various significance for the first and second stages of evaluation. The year and place were more significant for the first stage than for the second one. Performance group (dressage/jumping/eventing) influences the walk quality on both stages, as well as jumping and overall mean on the first. The country of origin was significant for the jumping trait and the final success on the first stage, and for jumping on the second stage. The age effect was significant only for the jumping trait on the first stage. Experience is needed for jumping, and it seems to be a more difficult trait. By taking into account the time of training (preparation period), the age effect becomes a non-significant effect. The training period was significant for two traits—canter evaluation and overall success. In all cases, regression on age showed that older horses had somewhat higher results, and the same was observed for training time.

The Spearman correlations (Table 3) showed that the results are somewhat unexpected. The same traits evaluated on these two stages were correlated as weak or moderate. The walk evaluations were correlated 0.43, the canter 0.45, and the trot 0.53. From the other side, all traits were much more correlated within stages, especially for the second stage, i.e., performance test training. Mean correlation for all calculated correlations between all traits for the second stage—performance field test—was 0.69. The same mean correlation for all traits evaluated on the first stage is much lower, 0.55 for performance traits and 0.58 when taking into account conformation traits, which are not evaluated on the second stage.

Even if the correlations are low or moderate, we calculate the mean between traits evaluated during these two stages/events to understand the overall skill ability. The created traits describe the same movement or jumping skills, even though they are evaluated without the rider on the first stage and under the rider on the second stage. The analysis of variance for the overall abilities, such as the overall gait or overall jumping skill, showed that the effects influencing the results are almost the same (Table 4 and Table 5). The year and place were significant for some traits; performance group and country of origin were the most important factors. As in the former analysis, the event (stage of evaluation) was more significant for the results than the age of the horses. The event was more significant for the results than the training period used earlier in the analysis of the second stage alone. It was surprising that all values for traits that differ between stages (trot, jumping, mean, and success) were higher in the first evaluation.

The calculations of the repeatability showed that this parameter is rather low for collected abilities. Except for the success measure (0.40), the values were below 0.3, and almost 0 for jumping. These values were expected to be higher (Table 5).

## 4. Discussion

Both genetic and environmental factors can influence the performance results. The analyses showed an important impact of the performance group and the country of origin. Many articles confirm the influence of origin or breed on performance results. Halo et al. [29] indicated a highly significant effect of breed as well as the proportion of genes (of Slovak Warmblood horses) on the results of horses that participated in performance tests and sports testing in the show jumping category of 4-, 5-, and 6-year-old young horses. In their research, a significant influence of the year of evaluation and the age of the horses was found. Our studies also confirmed the age effect on jumping traits, and the year × place effect on final assessment in the preselection stage, as well as the overall ability, such as the overall jumping skill. Age groups also differed according to Posta et al. [30] for some conformation and movement traits assessed during the performance test for Hungarian Sporthorse. The authors also analyzed the relationships between assessed traits. Free jumping and movement characteristics showed a low correlation compared to the average strength of associations in our research. High correlation was found between jumping ability traits in both studies. Low and moderate correlations were observed among other movement traits. Stewart et al. [22] confirmed the significant influence of age and discipline group (dressage, show-jumping, eventing) on the results of the British young horse test. The traits were scored lower for the dressage horse than ours, but in the correctness of gaits, they were rated higher than show jumping horses, but still lower than eventing horses. In their studies, genetic and environmental correlations between traits were generally high or moderate, and all were significant. Medium and high phenotypic as well as genetic correlations were found in research by Novotna et al. [31]. Positive and mostly high genetic correlations within and across traits of the broodmares’ inspections and mare performance test were found by Schopke et al. [32]. In our study, they were at a low-medium level, which probably indicates a high influence of environmental factors. The year and place effect incorporates the evaluator’s effect to some extent. Many studies show the influence of judges on ratings. In the research on the judges’ effect [33], based on the objective measurements, the judge effect was significant for the trait description.

Some differences between preselection and performance test results were caused by the rider effect. As mentioned, horses are usually scored only in free movement during qualifications, and in the final test, they are also assessed under the rider. The effect of the rider has been studied for many years [34,35,36,37,38,39]. Also, because of this, some countries included the rider effect in the statistical model for the breeding value estimation [40,41,42,43,44].

Analyses of the relationship between breeding test results at different stages of evaluation are extremely important. Bonow et al. [45] found that in the case of horses classified in the tests as jumping, 25 linear traits were significantly associated with show jumping results, and for dressage horses, 21 traits were significantly associated with competition results on the phenotypical level. The authors also pointed out significant differences between assessed traits for this performance group—jumping vs. dressage horses, which was also confirmed in our research for some traits. Comparable studies [46] were conducted in Poland, except for stallion performance tests after stationary riding training, which has not been taking place for several years. There was also a confirmed significant effect of the origin-breed group, age, as well as performance test place and year, on the assessed traits and the created indices at that event. The estimated rank correlations indicate some dependencies between scores on low and medium levels.

Recently, as mentioned above, stallions are assessed during the so-called field performance tests, also after a hundred days of training, but carried out in different places depending on the owner’s decision. This introduces additional environmental factors into the results, which is worth considering in future studies. These trials have been taking place since 2017, which creates further challenges for statistical and genetic analysis, as well as assessment of breeding value due to the small amount of data. The breeding value estimation is effective when the number of animals is large enough for detailed evaluation. It is usually assumed that at least 500 animals are necessary for detailed calculations of heritability. The animals have to be genetically linked, and the phenotype evaluation has to be realistic and detailed enough. There are possibilities for further improvement of the obtained datasets by making more frequent observations; however, these observations should be connected with each other and repeatable. A limitation of this study is connected to the amount of data. Even if the primary data file consisted of 334 stallions, only a part of them (23%) succeeded in entering and finishing the performance tests. The data cannot be easily collected, also because horses from different groups are not judged in all traits. Jumping horses are not evaluated in gaits, and dressage horses are not evaluated in jumping. Thus, the primary results should be confirmed on a larger dataset when available, especially if further genetic analysis would be possible. The calculations of the repeatability showed that this parameter is rather low for collected abilities. Except for the success measure (0.40), the values were below 0.3, and almost 0 for jumping. These values were expected to be higher.

Another interesting item addressed in this study was the dependencies between age and period of training. The structure of the data allowed for the investigation of the age and period of training at the same time. It allowed us to conclude that the effect of age being significant in the first selection stage for jumping trait was insignificant at the second stage in jumping evaluation, even for the more difficult traits like jumping under the rider. The development of the jumping technique was investigated based on the objective data [47,48,49]. Jumping technique was found to be individual, recognizable early in life, and not dependent on early foal training. Comparable conclusions were stated for the horse movement. The horse movement skills were found to be constant but individual for each horse on the basis of the repeatability and heritability of the walk and trot [50,51] and for jumping [52]. Therefore, it underlines the need for further analysis of the associations between horses’ movement scores at different stages of the training on different events: qualification and final test (after training session), as well as with the traits assessed in different conditions. Our results suggest that adding the period of training can be more significant for the horse’s performance than the age [53,54], even on such a young stage of performance as young horse breeding tests.

The obtained results, showing higher correlations between traits within the event, like correlations of the traits presenting the same ability, may suggest that the horse is evaluated by judges more as an overall current condition form, like the specific ability characteristics. The character of the evaluation, more optimistic at the earlier stage, is in accordance with the trends described in the former paper [55]. These aspects should be investigated further.

## 5. Conclusions

The horse data coming from different stages of stallion field performance tests, training, and horse presentation do not seem to be comparable. The connections between traits evaluated at different stages are of medium height (>0.3). The skills treated as the elements of the two stages do not correspond adequately with primary values. The repeatability of such evaluated skills is mostly low (<0.4). The training period is more important than age in the group of investigated young horses. Traits evaluated at every stage seem more connected, like traits of comparable skills between stages (<0.5). Traits after a longer period of training presented under the rider are more connected, like the same-named traits presented in a free-moving horse. The preliminary analysis does not allow for the data to be combined for breeding purposes. The obtained results showed that at least on the basic phenotypical level, the results of the same-named traits do not correspond strongly with each other. The study should be conducted further when genetic correlations would be available. Delay in performance test and longer training time showed, however, that this effect is significant and even more significant than the age of horses.

## Figures and Tables

**Table 1:** The characteristics of the evaluated traits and created abilities for all horses and years.

Trait		*n*	Mean	SD	Min	Max
Preselection stage	Walk	77	7.08	0.49	6.2	8.3
	Trot	77	7.64	0.48	6.2	8.5
	Canter	173	7.44	0.47	6.2	7.7
	Jumping	157	7.61	0.66	5.4	9.4
	Mean	180	7.54	0.47	5.9	6.5
	Success	180	0.76	0.43	0.0	1.0
Performance test stage	Walk	67	7.73	0.37	5.9	8.5
	Trot	67	7.02	0.56	5.5	6.1
	Canter	144	7.83	0.55	5.1	8.7
	Jumping	125	7.00	0.63	1.0	9.3
	Rideability	144	7.39	0.63	5.0	9.5
	Trainability	144	7.27	1.04	6.0	9.5
	Mean	144	7.36	0.74	4.5	8.9
	Success	144	0.56	0.49	0.0	1.0
Ability (both stages)	Walk	144	7.05	0.52	5.7	8.5
	Trot	144	7.35	0.64	5.5	8.9
	Canter	317	7.42	0.54	5.1	8.7
	Jumping	282	7.46	0.87	1.0	9.4
	Mean	280	7.54	0.62	4.5	8.9
	Success	324	0.67	0.47	0.0	1.0

**Table 2:** The statistically significant effects influencing the horse evaluations on different stages of performance tests (*p*-value; GLM; ne—not estimated).

Trait		Effects				
		Year × Place	Performance Group	Country of Origin	Age	Time of Training
		*p*-Value				
Preselection stage	Walk	-	0.0014	-	-	ne
	Trot	-	-	-	-	ne
	Canter	-	-	-	-	ne
	Jumping	-	<0.0001	0.0255	0.0374	ne
	Mean	0.0156	0.0003	-	-	ne
	Success	<0.0001	-	0.0266	-	ne
Performance test stage	Walk	-	0.0281	-	-	-
	Trot	-	-	-	-	-
	Canter	-	-	0.0297	-	0.0381
	Jumping	-	-	<0.0001	-	-
	Rideability	-	-	-	-	-
	Trainability	-	-	-	0.0913	-
	Mean	-	-	-	-	0.0940
	Success	-	-	-	-	0.0428

**Table 3:** Statistically significant results of Spearman correlations between different traits in both stages of training.

Traits		Preselection				Performance Test						
		Trot	Canter	Free Jumping	Points Overall	Walk	Trot	Canter	Jumping	Trainability	Rideability	Points Overall
		Correlations *p*-Value										
Preselection	Walk	0.30	-	-	0.33	0.43	-	-	-	-	-	-
		0.01			0.004	0.003						
	Trot		0.54	0.38	0.65	0.48	0.53	0.57	0.55	-	-	0.37
			0.0001	0.003	0.001	0.001	0.001	0.002	0.002			0.008
	Canter			0.57	0.63	0.40	0.63	0.45	0.22	-	0.20	0.23
				0.0001	0.001	0.007	0.001	0.001	0.03		0.04	0.02
	Jumping free				0.71	-	-	0.26	0.33	-	-	0.23
					0.001			0.01	0.001			0.03
	Points overall					0.30	0.33	0.29	0.27	-	-	0.21
						0.03	0.003	0.004	0.01			0.03
Performance Test	Walk						0.68	0.68	0.39	0.77	0.51	0.72
							0.001	0.001	0.006	0.001	0.001	0.0001
	Trot							0.80	0.51	0.82	0.52	0.73
								0.0001	0.0002	0.0001	0.001	0.0001
	Canter								0.80	0.71	0.53	0.86
									0.0001	0.001	0.0001	0.0001
	Jumping									0.69	0.51	0.88
										0.0001	0.0001	0.0001
	Trainability										0.69	0.84
											0.0001	0.0001
	Rideability											0.74
												0.0001

**Table 4:** The statistically significant effects of investigated factors on the created ability * (*p*-value; GLM/GLIMMIX).

Ability	Effects				
	Year × Place	Performance Group	Country of Origin	Age	Event
	*p*-Value				
Walk	-	<0.0001	-	-	-
Trot	-	0.0003	0.0178	-	<0.0001
Canter	-	-	0.0001	-	-
Jumping	-	0.016	0.08	-	0.0075
Mean	-	0.0346	-	-	0.0329
Success	0.02	-	0.03	-	0.07

* traits named the same on both stages of training were treated together.

**Table 5:** The statistically significant effects of investigated factors on the created ability * (*p*-value; MIXED/GLIMMIX).

Ability	Effects					
	Year × Place	Performance Group	Country of Origin	Age	Event	Repeatability
	*p*-Value					
Walk	-	<0.0001	-	-	-	0.23
Trot	-	0.0002	0.0521	-	<0.0001	0.17
Canter	-	-	0.0001	-	-	0.25
Jumping	0.07	0.0023	-	-	0.0074	0.03
Mean	-	0.0458	-	-	0.0288	0.06
Success	0.02	-	0.03	-	0.05	0.40

* traits named the same on both stages of training treated together.

## Data Availability

Data will be made available by the corresponding author upon reasonable request.

## Supplementary Materials

The following supporting information can be downloaded at: https://www.mdpi.com/article/10.3390/ani15223289/s1, Table S1. The numbers of horses on every stage of the training. Table S2. Regulations for preselection and performance test (according to the breeding programs of the Polish Horse Breeders Association, www.pzhk.pl).

## References

[1] Ducro B. (2011). Relevance of Test Information in Horse Breeding.

[2] Hellsten E.T., Viklund Å., Koenen E.P.C., Ricard A., Bruns E., Philipsson J. (2006). Review of genetic parameters estimated at stallion and young horse performance tests and their correlations with later results in dressage and show-jumping competition. Livest. Sci..

[3] Thorén H.E. (2008). International Sport Horse Data for Genetic Evaluation. Ph.D. Thesis.

[4] Koenen E.P.C., Aldridge L.I., Philipsson J. (2004). An overview of breeding objectives for warmblood sport horses. Livest. Prod. Sci..

[5] Ruhlmann C., Janssens S., Philipsson J., Thorén-Hellsten E., Crolly H., Quinn K., Manfredi E., Ricard A. (2009). Genetic correlations between horse show jumping competition traits in five European countries. Livest. Sci..

[6] Van Barneveld A.J., Dijkstra J., Van der Werf J.H. (2021). The impact of genomic selection on breeding values for athletic traits in Dutch Warmblood horses. Anim. Genet..

[7] Van Barneveld A.J., Dijkstra J. (2022). Estimation of breeding values using genomic information: The Dutch experience. Anim. Genet..

[8] Knapp W., Ludwig M. (2022). Genomic selection in German horse breeding. Anim. Genet..

[9] Lewczuk D. (2015). Effect of the age on performance tests in Warmblood horses in Poland. J. Vet. Behav..

[10] Lewczuk D. (2020). Toward the best young horse performance—Variability of behavior and performance-related traits in Polish warmbloods during years 2002–2017. J. Vet. Behav..

[11] Viklund Å., Näsholm A., Strandberg E., Philipsson J. (2011). Genetic trends for performance of Swedish Warmblood horses. Livest. Sci..

[12] Ducro B.J., Koenen E.P.C., Van Tartwijk J.M.F.M., Bovenhuis H. (2007). Genetic relations of movement and free-jumping traits with dressage and show-jumping performance in competition of Dutch Warmblood horses. Livest. Sci..

[13] Bonow S., Eriksson S., Thorén Hellsten E., Gelinder Viklund Å. (2023). Consequences of specialized breeding in the Swedish Warmblood horse population. J. Anim. Breed. Genet..

[14] Bonow S. (2023). Breeding of Swedish Warmblood Horses towards Specialization in Show Jumping and Dressage.

[15] Dubois C., Ricard A. (2007). Efficiency of past selection of the French Sport Horse: Selle Français breed and suggestions for the future. Livest. Sci..

[16] Haberland A.M., von Borstel U.K., Simianer H., König S. (2012). Integration of genomic information into sport horse breeding programs for optimization of accuracy of selection. Animal.

[17] Bellone R.R., Avila F. (2020). Genetic testing in the horse. Vet. Clin. Equine Pract..

[18] Raudsepp T., Finno C.J., Bellone R.R., Petersen J.L. (2019). Ten years of the horse reference genome: Insights into equine biology, domestication and population dynamics in the post-genome era. Anim. Genet..

[19] Palmer E., Chavatte-Palmer P. (2020). Contribution of reproduction management and technologies to genetic progress in horse breeding. J. Equine Vet. Sci..

[20] Bartolomé E., Cervantes I., Gómez M.D., Molina A., Valera M. (2008). Influence of environmental factors on the sport performance of the horse, in an objective selection test (Show Jumping). ITEA.

[21] Posta J., Komlósi I., Mihók S. (2009). Breeding value estimation in the Hungarian Sport Horse population. Vet. J..

[22] Stewart I.D., Woolliams J.A., Brotherstone S. (2011). Genetic evaluations of traits recorded in British young horse tests. Arch. Anim. Breed..

[23] Kubištová B., Jiskrová I. (2017). Some Effects on the Performance of the Czech Warm-Blood Horse in the Horse Breeding Station (ŠCHK)-Měník. Acta Univ. Agric. Silvic. Mendel. Brun..

[24] Solé M., Bartolomé E., Sánchez M.J., Molina A., Valera M. (2017). Predictability of adult Show Jumping ability from early information: Alternative selection strategies in the Spanish Sport Horse population. Livest. Sci..

[25] Solé M., Sánchez M.J., Valera M., Molina A., Azor P.J., Sölkner J., Mészáros G. (2017). Assessment of sportive longevity in Pura Raza Español dressage horses. Livest. Sci..

[26] Solé M., Valera M., Fernández J. (2018). Genetic structure and connectivity analysis in a large domestic livestock meta-population: The case of the Pura Raza Español horses. J. Anim. Breed. Genet..

[27] Sobotková E., Mikule V., Kuřitková D., Jiskrová I., Sládek L. (2022). Analysis of the current situation in international show jumping and assessment of the influence of the proportion of Thoroughbred in the pedigree, horse demographics and sport season on the performance of horses. J. Vet. Behav..

[28] Giontella A., Silvestrelli M., Cocciolone A., Pieramati C., Sarti F.M. (2024). Breeding value Estimation based on morphological evaluation of the Maremmano horse population through factor analysis. Animals.

[29] Halo M., Mlyneková E., Massányi M., Solár D., Halo Jr M., Moravčíková N. (2021). Influence of indirect factors and its effect analysis on performance level of Slovak warmblood horse breed. Acta Fytotechn. Zootech..

[30] Posta J., Komlósi I. (2007). Genetic analysis of selected body measurements of Hungarian Sport Horse mares. Acta Agrar. Debreceniensis.

[31] Novotna A., Birovas A., Vostra-Vydrova H., Vesela Z., Vostry L. (2022). Genetic parameters of performance and conformation traits of 3-year-old warmblood sport horses in the Czech republic. Animals.

[32] Schöpke K., Wensch-Dorendorf M., Swalve H.H. (2013). Genetic evaluations of the German Sport Horse: Population structure and use of data from foal and mare inspections and performance tests of mares. Arch. Anim. Breed..

[33] Lewczuk D. (2013). Effect of the judge and definition of the trait for horse free jumping evaluation. Arch. Anim. Breed..

[34] Oki H., Sasaki Y., Willham R.L. (1994). Genetics of racing performance in the Japanese Thoroughbred horse: II. Environmental variation of racing time on turf and dirt tracks and the influence of sex, age, and weight carried on racing time. J. Anim. Breed. Genet..

[35] Powers P., Harrison A. (2002). Show-Jumping: Effects of the rider on the linear kinematics of jumping horses. Sports Biomech..

[36] Powers P.N.R., Harrison A.J. (2004). Influences of a rider on the rotation of the horse–rider system during jumping. Equine Comp. Exerc. Physiol..

[37] Powers P.N., Kavanagh A.M. (2005). Effect of rider experience on the jumping kinematics of riding horses. Equine Comp. Exerc. Physiol..

[38] Hanousek K., Salavati M., Dunkel B. (2020). The impact of horse age, sex, and number of riders on horse performance in British eventing horse trials. J. Equine Vet. Sci..

[39] Neumann C., Čítek J., Janošíková M., Doležalová J., Starostová L., Stupka R. (2021). Effects of horse age and the number of riders on equine competitive performance. J. Vet. Behav..

[40] Sánchez Guerrero M.J., Cervantes I., Valera M., Gutiérrez J.P. (2014). Modelling genetic evaluation for dressage in Pura Raza Español horses with focus on the rider effect. J. Anim. Breed. Genet..

[41] Stewart I.D., White I.M.S., Gilmour A.R., Thompson R., Woolliams J.A., Brotherstone S. (2012). Estimating variance components and predicting breeding values for eventing disciplines and grades in sport horses. Animal.

[42] Posta J., Mihok S., Markus S., Komlosi I. (2009). Analysis of Hungarian sport horse show jumping results using different transformations and models. Arch. Anim. Breed..

[43] Novotná A., Bauer J., Vostrý L., Jiskrová I. (2014). Single-trait and multi-trait prediction of breeding values for show-jumping performance of horses in the Czech Republic. Livest. Sci..

[44] Bartolomé E., Menéndez-Buxadera A., Molina A., Valera M. (2018). Plasticity effect of rider–horse interaction on genetic evaluations for Show Jumping discipline in sport horses. J. Anim. Breed. Genet..

[45] Bonow S., Eriksson S., Strandberg E., Hellsten E.T., Viklund Å.G. (2024). Phenotypic associations between linearly scored traits and sport performance in the Swedish Warmblood horse population. Livest. Sci..

[46] Pietrzak S., Próchniak T., Kozak-Jurek K., Zapala A. (2016). Effect of some factors on performance value assessment of stallions during performance tests. Ann. Anim. Sci..

[47] Santamaría S., Bobbert M.F., Back W., Barneveld A., van Weeren P.R. (2004). Evaluation of consistency of jumping technique in horses between the ages of 6 months and 4 years. Am. J. Vet. Res..

[48] Santamaría S., Bobbert M.F., Back W., Barneveld A., van Weeren P.R. (2004). Variation in free jumping technique within and among horses with little experience in show jumping. Am. J. Vet. Res..

[49] Santamaría S., Bobbert M.F., Back W., Barneveld A.B., van Weeren P.R. (2005). Effect of early training on the jumping technique of horses. Am. J. Vet. Res..

[50] Valera M., Galisteo A.M., Molina A., Miró F., Gómez M.D., Cano M.R., Agüera E. (2008). Genetic parameters of biokinematic variables of the trot in Spanish Purebred horses under experimental treadmill conditions. Vet. J..

[51] Molina A., Valera M., Galisteo A.M., Vivo J., Gómez M.D., Rodero A., Agüera E. (2008). Genetic parameters of biokinematic variables at walk in the Spanish Purebred (Andalusian) horse using experimental treadmill records. Livest. Sci..

[52] Lewczuk D. (2017). Some remarks on repeatability and heritability of the bascule technique in jumping horses. J. Equine Vet. Sci..

[53] McKeever K.H. (2002). Exercise physiology of the older horse. Vet. Clin. Equine Pract..

[54] McKeever K.H. (2003). Aging and how it affects the physiological response to exercise in the horse. Clin. Tech. Equine Pract..

[55] Wejer J., Lewczuk D. (2016). Effect of the age on the evaluation of horse conformation and movement. Ann. Anim. Sci..

