# Supplementary material for: Comparative Analysis of the Stallion Field Performance Test at Different Training Stages and Horse Age
Source: Animals (Basel). 2025 Nov 13;15(22):3289. doi: 10.3390/ani15223289 (PMC12649309; doi:10.3390/ani15223289)
Supplement: Supplementary file 1 [file animals-15-03289-s001.zip › animals-3943314-supplementary.pdf]

Table S1. The numbers of horses on every stage of the training

Year of qualification	Horses (numbers)					Performance group according to qualifications year (available for 300 horses) *			Country of origin according to qualifications year (available for 299 horses) *								Age at qualification in days	Age at performance test in days	Training time in days
	preselection			Performance tests													mean (SD)	mean (SD)	mean (sd)
	Presented for conformation	Qualified for performance preselection	Succeed for test	tested	succeed	dressage	jumping	eventing	Belgium	Danemark	The Netherlands	Ireland	Germany	Poland	Sweden	Great Britain			
**2017**	16	16	16	12	9	0	12	3	0	0	2	0	1	12	0	0	1165 (133)	1307 (131)	142 (3)
**2018**	53	25	25	15	11	3	36	12	2	0	5	0	1	37	0	0	1179 (157)	1338 (157)	159 (0)
**2019**	64	22	22	-	-	2	40	14	3	0	3	2	8	38	0	1	1170 (290)	-	-
**2020**	55	35	16	18	10	4	45	6	1	0	1	0	7	39	1	0	916 (93)	1711 (277)	541 (59)
**2021**	70	27	27	59	31	10	46	14	2	1	1	0	7	47	0	1	1015 (110)	1345 (105)	373 (70)
**2022**	76	56	31	32	20	7	47	9	0	0	2	0	13	60	0	1	1059 (110)	1341 (73)	255 (0)
**total**	**334**	**180**	**144**	**136**	**81**	**26**	**226**	**58**	**8**	**1**	**14**	**2**	**37**	**233**	**1**	**3**	**1164 (320)**	**1436 (339)**	**346 (139)**

* the data do not always sum into 334 because of the missing values

Table S2. Regulations for preselection and performance test (according to the breeding programs of the Polish Horse Breeders Association - www.pzhk.pl)

TRAIT		definition	conditions	evaluators
**Preselection stage**	**Basic rules**	Qualifications min. 30 months, but until the end of third year of age<br>**Group A**<br>(dressage an Trakhenian horses pedigree) – qualified if mean min. 7,5 points from: walk, trot i canter presented free, no trait can be below 6.8 points<br>**Grup B**<br>(jumping stallions) –<br>qualified if mean min. 7,5 points from: free canter and free jumping, free jumping cannot be below 7.5 points<br>**Grup C** (Trakhenian Stallions and Stallions with 50% of Thoroughbred, Arabian and Anglo-Arabian pedigree, or Maloplski and Wielkopolski from the endangered part of the breed) - qualified if mean min. 7,2 points from: free jumping, walk, trot i canter presented free, no trait can be below 6.0 points	Training centre with riding hall, qualified people for free horse movement management.<br><br>Before the stallion begins the movement and free jumping assessment, it must be warmed-up and then guided by the staff into a figure of eight, first at a canter, then at a trot. After the jumps in the hall are completed, the jury evaluates the walk during the presentation of the stallion leading in hand. The figure of eight movement allows for the assessment of the stallion's balance, reaction speed, and agility.	Judges committee appointed by Polish horse Breeders Association.<br>Horse can be withdrawn at any time by riders or judges.<br><br>Stallions that did not obtain the minimum marks for type or conformation (min. 7.5 points for conformation and 7.5 points for type or 7.0 in the case of endangered part) will not be allowed to participate in the next parts of the test.
	**Walk**	The following elements are assessed: energy, stride length, regularity, relaxation, joint	The horse should be led "in hand" along the long wall of the riding hall .	Judges committee appointed by Polish horse Breeders Association.

		flexibility, back and neck movement and engagement of the hind legs..		Horse can be withdrawn at any time by riders or judges.
	**Trot**	The following elements are assessed: energy, length of stride, regularity, work of the hindquarters and range of the limbs movement, suppleness, work of the back and ne	See basic rules above	Judges committee appointed by Polish horse Breeders Association.<br>Horse can be withdrawn at any time by riders or judges.
	**Canter**	The following elements are assessed: energy, regularity, lightness of the front, engagement of the hindquarters, suppleness, flexibility of the joints, work of the back and neck.	See basic rules above	Judges committee appointed by Polish horse Breeders Association.<br>Horse can be withdrawn at any time by riders or judges.
	**Jumping**	The following elements are assessed: energy, regularity, lightness of the front, engagement of the hindquarters, suppleness, flexibility of the joints, and the work of the back and neck.<br><br>The following elements are also assessed: carefulness by approach to the obstacle, head and neck position by take-off, power of take-off and speed of foreleg lift, position of the head, neck, and body during the jump (baskil), ability to flex the forequarters and open the hindquarters, evenness of the	In the riding hall min. 20x40 m. jumping line is placed along the long wall. The stallion moves on left and it starts from the corner by cantering. Jumping starts from the guide rail 3m before vertical fence. In the distance 6.5-7.2 the doublebarre obstacle is placed. The height and organization of jumps depend on the judging commission. In exceptional cases, the stallion may be released in a "hand-held" combination.	Judges committee appointed by Polish horse Breeders Association.<br>Horse can be withdrawn at any time by riders or judges.

		forequarters, dynamics of the jump, suppleness of the back, and speed of recovery after the jump.		
Performance test stage	**Basic rules**	After at least 100 days of training Regulations for the groups A,B,C as above	Adequate, certified by Polish Horse Breeders Association training centre with the qualified trainers and people	Judges committee appointed by Polish horse Breeders Association.<br>Horse can be withdrawn at any time by riders or judges.
	**Walk**	elements assessed: energy, stride length, regularity	Group A - Dressage Stallions - presentation before the judging panel. The stallion is presented and evaluated by the test rider. The judging panel issues instructions regarding the figures performed in the arena. There is no dressage program..	Judges committee appointed by Polish horse Breeders Association.<br>Horse can be withdrawn at any time by riders or judges.
	**Trot**	elements assessed: energy, stride length, regularity	As above	Judges committee appointed by Polish horse Breeders Association.<br>Horse can be withdrawn at any time by riders or judges.
	**Canter**	elements assessed: energy, regularity, lightness of the front, engagement of the hindquarters	As above	Judges committee appointed by Polish horse Breeders Association.<br>Horse can be withdrawn at any time by riders or judges.
	**Jumping**	elements assessed: jumping style, jumping ease, reflexes, jumping capabilities, courage	Runs of jumps elements (four obstacles in the riding hall, two in the line, two single) in the following runs and orders:<br>1. vertical obstacle (60 cm) – max. 2 runs<br><br>2. vertical obstacle (90 cm) and second oxer (ok. 90 cm x 90 cm) – max. 2 runs	Judges committee appointed by Polish horse Breeders Association.<br>Horse can be withdrawn at any time by riders or judges.

			3. vertical obstacle (90 cm), , oxer (90 cm x 90 cm), then vertical (80 cm) and in the distance 10.5-11m oxer (100 cm x 100 cm) – max. 2 runs<br><br>4. vertical obstacle (ok. 100 cm), oxer (100 cm x 100 cm), vertical (90cm) and in the distance 10.5-11m oxer (110 cm x 110 cm) – max. 3 runs<br><br>Horses of the C group jump 10 cm lower.<br>Horses jump from the canter, the warming-up is outside the riding hall.	
	**Rideability**	elements assessed: willingness to cooperate with the rider, submission to the rider's will, susceptibility to aids, softness of "carrying" and the rider's well-being during rides	Before the evaluation by a third-party rider, stallions are warmed up by a rider designated by the owner. During the preparation of a stallion in the warm-up arena, the same rules apply as for the evaluation of a horse during the trial (including riding equipment, obstacle height, and the number of jumps). The test riders evaluate: showjumpers – showjumping stallions (B); dressage riders – dressage stallions (A); and eventers – stallions from Group C.	Equestrian Federation sport events, according to the discipline the experience with young horses
	**Trainability**	Usability for special sport discipline – for dressage in A group, for jumping for B group and eventing for C group .		Riders competing in the Polish Equestrian Federation sport events, according to the discipline the experience with young horses
